# Non-specificity of sequence characterised amplified region as an alternative molecular epidemiology marker for the identification of *Salmonella enterica* subspecies *enterica* serovar Typhi

**DOI:** 10.1186/s13104-018-3870-z

**Published:** 2018-10-29

**Authors:** Ja’afar Nuhu Ja’afar, Subhash Janardhan Bhore, Kia Kien Phua

**Affiliations:** 1 Enteric Diseases Research Cluster, Institute for Research in Molecular Medicine (INFORMM), Universiti Sains Malaysia, 11800, USM George Town, Penang Malaysia; 2 Department of Biotechnology, School of Life Sciences, Modibbo Adama University of Technology (MAUTECH), Yola, PMB 2076 Adamawa State Nigeria; 3 Department of Biotechnology, Faculty of Applied Sciences, AIMST University, 08100 Bedong, Kedah Malaysia

**Keywords:** SCAR, RAPD, *S*. Typhi, Kelantan, Malaysia

## Abstract

**Objective:**

Identification of *Salmonella* Typhi by conventional culture techniques is labour-intensive, time consuming, and lack sensitivity and specificity unlike high-throughput epidemiological markers that are highly specific but are not affordable for low-resource settings. SCAR, obtained from RAPD technique, is an affordable, reliable and reproducible method for developing genetic markers. Hence, this study investigated the use of SCAR as an alternative molecular epidemiological marker for easy identification of *S.* Typhi in low-resource settings.

**Results:**

One hundred and twenty RAPD primers were screened through RAPD-PCR against a panel of common *enterobacteriaceae* for the best RAPD band pattern discrimination to develop SCAR primers that were used to develop a RAPD-SCAR PCR. Of this number, 10 were selected based on their calculated indices of discrimination. Four RAPD primers, SBSA02, SBSA03, SBSD08 and SBSD11 produced suitable bands ranging from 900 to 2500 bp. However, only SBSD11 was found to be specific for *S.* Typhi, and was cloned, sequenced and used to design new SCAR primers. The primers were used to amplify a panel of organisms to evaluate its specificity. However, the amplified regions were similar to other non-Typhi genomes denoting a lack of specificity of the primers as a marker for *S.* Typhi.

## Introduction

Typhoid fever, a systemic disease caused by the bacterium *Salmonella enterica* subspecies *enterica* serovar Typhi (*S.* Typhi), is global in distribution but more prevalent in Oceania, Africa, Latin America and Asia with prevalence rates of 15.4, 49.8, 53.1, and 274.3 per 100,000 population, respectively [1].

For *S.* Typhi identification and genotyping, conventional culture techniques are labour intensive, time consuming, expensive, and lack sensitivity and specificity [2, 3]. In fact, epidemiologically unrelated *S.* Typhi isolates are often so similar and look identical using most typing techniques [4]. On average, an estimated time span of 4–7 days is required to obtain a positive result, excluding the time for serotyping [5, 6]. Currently, high throughput epidemiological markers such as pulse-field gel electrophoresis (PFGE) and single nucleotide polymorphism (SNP) markers are employed to track and monitor *S.* Typhi and the disease it causes [7–9]. However, these markers are expensive to develop and are not readily affordable in low-resource settings where the disease is mostly endemic.

Random amplified polymorphic DNA-PCR (RAPD-PCR) is a rapid and sensitive PCR method that enables the amplification of independent genetic loci of the target genome. It has been developed for genetic mapping, fingerprinting, and is widely used in inter- and intra- specific population polymorphism analyses of different organisms [10]. It has proved to be a powerful tool for discriminating different species or subspecies of organisms, and for genetic analysis or phylogenetic relationships among strains for a variety of microorganisms, plants, and mammals [11, 12]. In addition, it has been used for strain discrimination in various *S. enterica* serovars [13–16]. However, this method has an underlying disadvantage of being less reliable due to its sensitivity to reaction parameters such as quality of DNA template, concentrations of PCR components and PCR cycling conditions [17, 18]. Sequence characterised amplified region (SCAR) is derived by converting RAPD markers through cloning and sequencing the two ends of the amplified polymorphic RAPD fragments [19]. SCAR markers are more reliable, efficient and advantageous than RAPD markers because they are reproducible, less sensitive to reaction parameters and able to detect a single locus. Hence, these qualities allowed its use as a genetic marker [19–21].

Because of the known irreproducible nature of RAPD-PCR as a genotyping method within the scientific community, RAPD-SCAR using a pair of specific oligonucleotide primers derived from RAPD-PCR was explored. Although successfully used in plant and animal studies [22–26], the technique has been transferred to bacteria with varying degrees of successes. Hence, the present study was designed to investigate the possibility of developing SCAR marker as an alternative epidemiological marker for easy identification of *S.* Typhi in low-resource settings.

## Main text

### Methods

#### Bacteria isolates

A panel of 26 genomic DNA samples were used in this study. Sixteen *S.* Typhi isolates, previously differentiated by pulsed-field gel electrophoresis (PFGE) and differing in district and year of isolation [27, 28], were obtained from Hospital Universiti Sains Malaysia (HUSM), Kubang Kerian, Kelantan. The other ten isolates were either purchased from the American Type Culture Collection (ATCC) or obtained from the Biobank of the Institute for Research in Molecular Medicine (INFORMM), USM, Kelantan. These include *Salmonella* Paratyphi A (ATCC 9150), *Salmonella* Paratyphi B (ATCC BAA 1250), *Salmonella* Paratyphi C (ATCC 9068), *Salmonella* Typhimurium (ATCC 14028), *Salmonella* Poona (ATCC 04840), *Salmonella* Enteritidis (ATCC 13076), *Shigella sonnei*, *Yersinia enterocolitica*, *Klebsiella pneumoniae* and *Escherichia coli*.

#### Genomic DNA extraction

This was achieved using QIAGEN^®^ DNA extraction kit, (DNeasy^®^ Blood and Tissue Kit, USA). DNA concentration was measured using nanodrop (NANODROP 2000c, USA).

#### RAPD primers

Six kits of RAPD primers, (SBS A-F from SBS Genetech Co., Ltd. China), containing 20-decamer oligonucleotides each, were used. The primers have melting temperatures (Tm) of either 32 °C or 34 °C with a GC content of 60% or 70%, respectively.

#### Screening and selection of RAPD primers

All six kits (SBS A-F) were screened to select primers that have the best pattern discrimination. The optimized RAPD-PCR method [29] and three random *S.* Typhi isolates that had been previously differentiated using PFGE were used for the screening. A criterion was set by calculating the index of discrimination, defined as the ratio of maximum number of bands to minimum number of bands for the three isolates. Furthermore, an additional criterion was set for primers with the least ratio scores, which is “a primer with least ratio score that has higher maximum number of bands will be selected over a primer with lower maximum number of bands”. Therefore, 10 primers were selected for subsequent RAPD-PCR screening of the 26 isolates.

#### RAPD-PCR assay

The optimised RAPD-PCR method of Ja’afar et al. [29] was adopted while the method of Melotto et al. [30] was adopted for SCAR marker development.

#### Gel purification, cloning and sequencing of RAPD-PCR product

Clear RAPD bands that were only present in *S.* Typhi were gel-purified using QIAGEN^®^ gel extraction kit (QIAquick^®^ Gel Extraction Kit, USA) and cloned using QIAGEN^®^ cloning kit (QIAGEN^®^ PCR Cloning^*plus*^ Kit, USA). Plasmids of positive clones were extracted using QIAGEN^®^ plasmid extraction kit (QIAprep^®^ Spin Miniprep Kit, USA) and sent to First BASE Laboratories Sdn. Bhd., Malaysia for sequencing.

#### Primer design and validation of SCAR primers

Sequence homology search for each sequence was performed within GenBank’s database [31] and unique primers were designed, synthesized and validated.

### Results

#### Selected RAPD primers

**Fig. 1:**
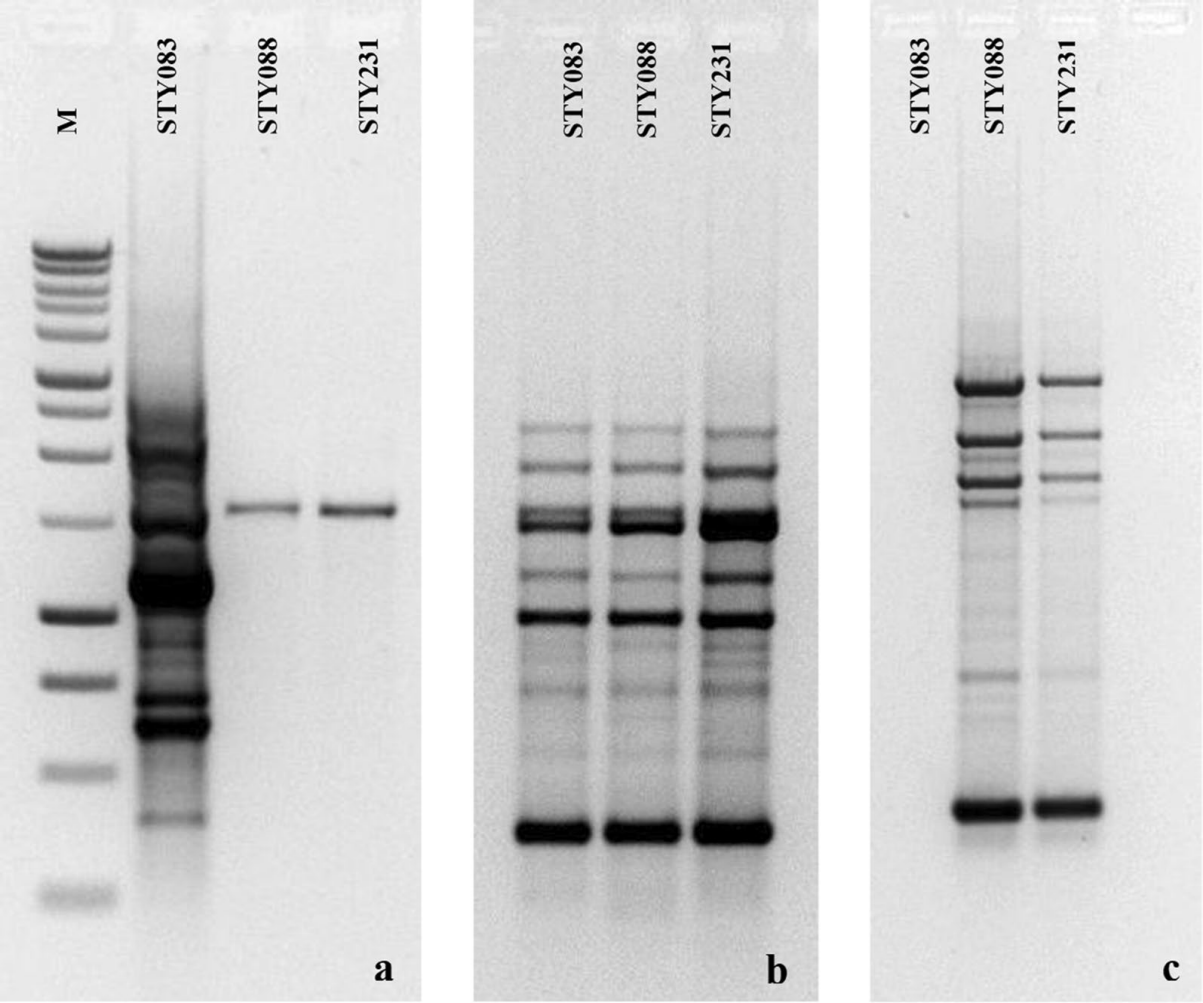
Example of scoring for different primers. Primers had both high and low bands in at least two isolates (**a**); equal number of bands in all isolates (**b**); no amplification in at least one isolate (**c**). M: Marker; STY083, STY088 and STY231: *S*. Typhi isolates

The number of PCR bands was based on amplification of three selected *S.* Typhi isolates (STY083 (ATCC 7251), STY088 and STY231) that had been previously differentiated by pulsed-field gel electrophoresis (PFGE). Primers with high ratio scores had both high and low number of bands in at least two of the three isolates (Fig. 1a). It does not mean that the primer yielded higher number of bands in all isolates. In fact, primers with higher or equal number of bands in all isolates had low ratio scores (Fig. 1b). Conversely, primers with least ratio scores had no amplification in at least one of the isolates (Fig. 1c). In such instance, they were scored for the isolate(s) that had at least one band only.

#### RAPD-PCR bands selection

**Fig. 2:**
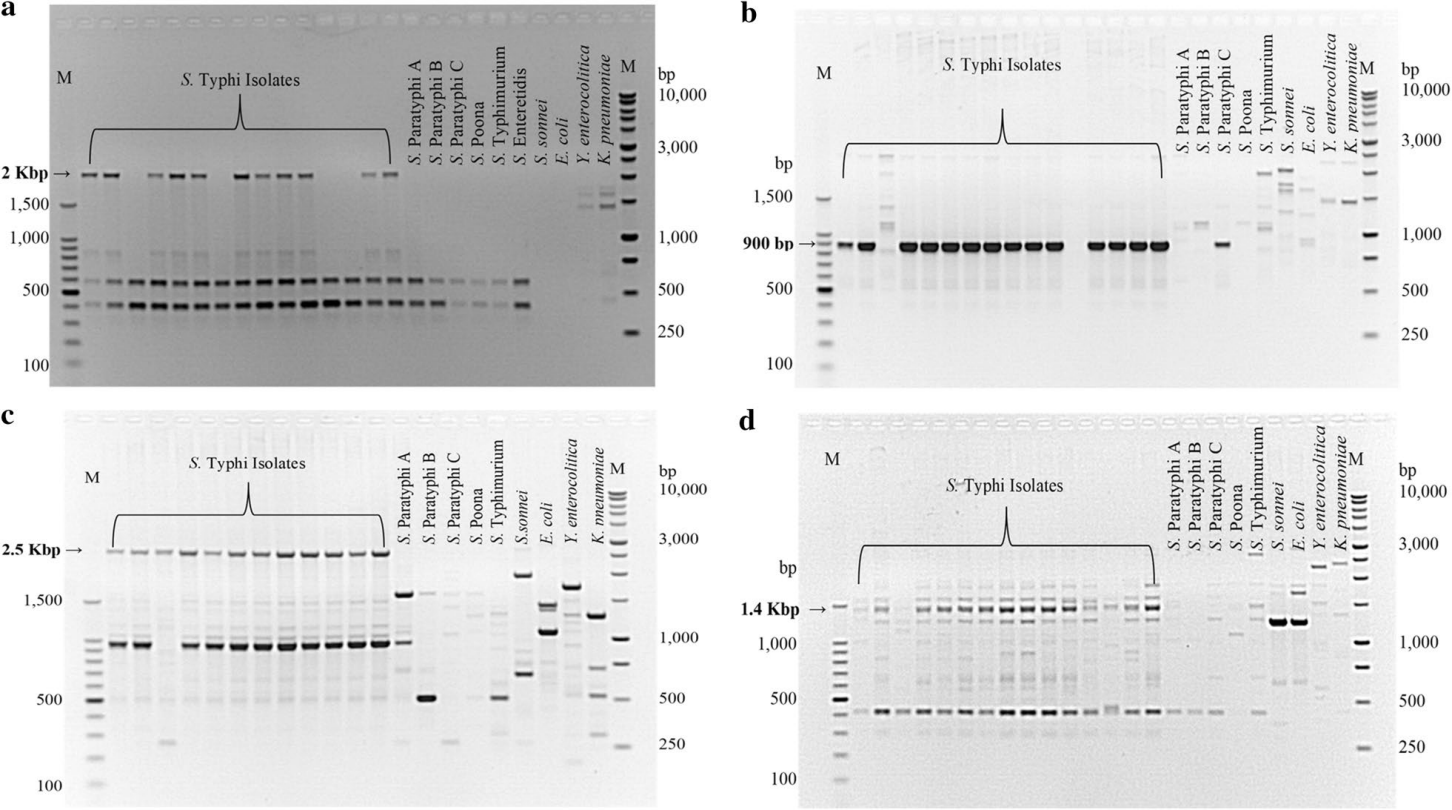
RAPD primers showing suitable bands for SCAR marker development. **a** Primer SBSA02 showing a 2 Kbp band found only amongst *S*. Typhi isolates. **b** Primer SBSA03 showing a 900 bp band found in both *S*. Typhi, *S.* Paratyphi C and *E. coli*. **c** Primer SBSD08 showing a 2.5 Kbp band found only in *S.* Typhi. **d** Primer SBSD11 showing a 1.4 Kbp band found in both *S*. Typhi, *S.* Typhimurium and *Y. enterocolitica*. M: 100 bp and 1 Kbp ladders, respectively

Out of the ten RAPD primers, four (SBSA02, A03, D08 and D11) produced suitable bands (ranging from 900 to 2500 bp) for SCAR marker development (Fig. 2a–d).

#### RAPD-PCR product cloning

Eluted DNA of the three *S.* Typhi isolates from each of the four primers above (12 in total) were used for cloning reactions. Only the three DNA products from primer SBSD11 that produced positive clones were sent for sequencing.

#### Primer design for SCAR markers

Sequencing results from both T7 and SP6 promoter regions of the pDrvie plasmid provided the sequence composition of the cloned DNA fragment. The sequences were bioinformatically stringed using MEGA software (Version 5.2, [32]). Blasting the sequence against the non-redundant database of NCBI showed that it encodes a Type IV secretory pathway protein, virB4 component and a lipoprotein in *S.* Typhi and a hypothetical protein in *S.* Typhimurium.

#### Validation of SCAR primers

**Fig. 3:**
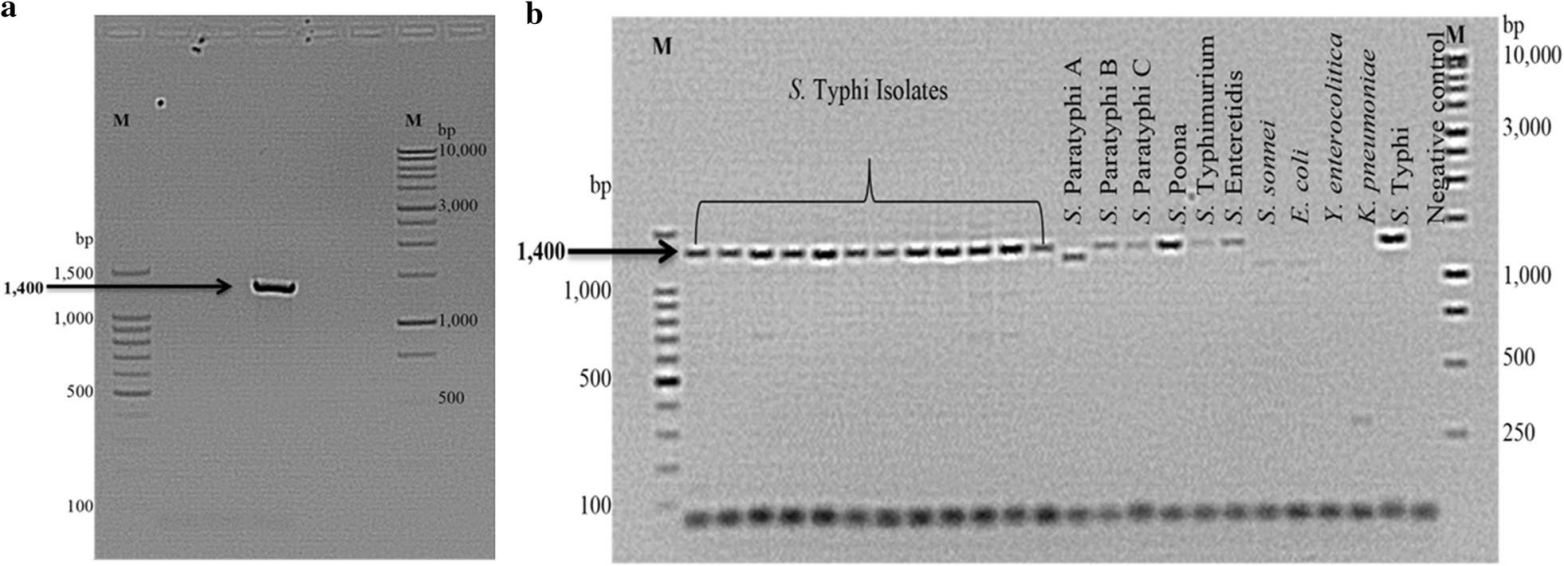
**a** Optimized SCAR-PCR assay using recombinant pDrive plasmid as DNA template. Optimum annealing temperature was 56 °C. M: 100 bp and 1 Kbp ladders, respectively. **b** Gel showing results of PCR assay using specific SCAR primer. M: 100 bp and 1 Kbp ladders, respectively

The recombinant pDrive plasmid was used as template DNA to optimize both annealing temperature and PCR reaction conditions of the synthesized primers (Fig. 3a). Following optimization, the primer was used to amplify the panel of organisms for confirmation. However, the amplified regions were similar to other non-Typhi genomes denoting lack of specificity of the primer as a marker for *S.* Typhi (Fig. 3b).

### Discussion

In an effort to develop a simple, fast and cost effective molecular epidemiology marker for identifying *S.* Typhi from other *Salmonella* species, 120 random primers were screened through RAPD-PCR against a panel of common *enterobacteriaceae* to develop SCAR primers that were used for RAPD-SCAR PCR.

During RAPD primer screening, some inconsistent amplifications were observed. In some instances, no bands were seen for certain primers, while some generate complex band patterns that were difficult to interpret. These sorts of inconsistencies have been reported previously whereby no amplification, difficult to interpret complex patterns, and primer artefacts, were observed [33, 34]. More so, lack of amplification observed with some primers could be attributed, theoretically, to the distance of the primers on the 5′ and 3′ directions on the template DNA. A distance of more than 4 kbp between the primers on the 5′ and 3′ directions has been reported to result in no amplification [35].

RAPD-PCR screening of bands for SCAR marker development was done using four primers, SBS-A02, -A03, -D08 and -D11, with suitable bands ranging from 900–2500 bp (Fig. 2a–d). Following cloning, only primer SBSD11 produced positive clones that were sent for sequencing. A specific SCAR primer set was designed for this sequenced fragment. The SCAR primer had, in addition, the original RAPD primer sequence in order to confirm the fragment’s specificity to *S.* Typhi. After PCR optimization with the SCAR primer (Fig. 3a), another PCR was performed on the same panel of bacteria (Fig. 3b). The procedure was successful as it was able to identify all *S.* Typhi isolates. However, other subspecies *enterica* were also amplified though non-*salmonella* isolates such as *E. coli*, *Y. enterocolitica* and *K. pneumoniae* were not amplified (Fig. 3b). Inference to this phenomenon was drawn through published literatures. First, it could be explained by the fact that the isolates tested were all from the same subspecies, *enterica*, and that they share similar genetic content [36]. It has been reported that the serovars Typhi and Typhimurium share genetic homology in important pathogenicity elements [37]. Similarly, Parkhill et al. [38] has demonstrated that of the 204 pseudogenes present in *S.* Typhi, 75 of them were involved in housekeeping functions in other serovars. Pseudogenes are genes that have lost functions due to insertions, deletions or substitutions [38]. Furthermore, Chan et al. [39] have shown the close relationship of serovar Typhi to serovars Paratyphi A and Sendai in a microarray study. In the same manner, high similarity in gene contents has been reported for serovar Typhi strain CT18 and serovar Paratyphi A strain ATCC 9150 [40]. Therefore, the SCAR primer designed in this study may anneal to target sequences found in closely related serovars. Affirmatively, Aksoy [41], when identifying SCAR markers for *S.* Typhimurium, reported similar findings to this work. In the study, the 700 bp band found to be specific to *S.* Typhimurium by RAPD-PCR could not be used for further studies.

However, successful use of SCAR markers has been reported for *Trypanosoma cruzi* [42] most probably due to the conservative nature of its genome and for the analysis of genomic instability in breast cancer tissues [43]. Other successful applications of SCAR markers have been for the detection of *Agrobacterium vitis* in rice [44], *Pseudomonas brassicacearum* as a biological control agent of snow mould in winter wheat [20] and *Xylella fastidiosa* in grape vine disease [45]. Similarly, it has been utilised in the identification of strawberry genotypes carrying red stele resistance gene for mass breeding [46, 47], preservation of an endangered ornamental tree species [48] and for adulteration detection [49, 50].

### Conclusion

Although the SCAR marker developed in this study to specifically identify *S.* Typhi was successful, yet other serovars of the subspecies *enterica* were also amplified, suggesting the limited specificity of SCAR markers as alternative to the gold-standard, PFGE, in the identification of *S.* Typhi. However, the marker developed could instead, be used as a preliminary screening tool for *Salmonella enterica* subspecies rather than identifying a specific *Salmonella* serovar due to homologous nature of their genomes. More so, more research on this topic needs to be done to preclude the use of SCAR markers in *Salmonella* species.

## Limitations

The need for stringent thermocycling conditions for RAPD assay optimization, limits the speed of SCAR development for *S.* Typhi even though the assay is low cost. Similarly, laboratory differentiation of isolates in closely related species, such as *Salmonella enterica*, is difficult due to sequence homology of their genomes.

## Abbreviations

SCAR: sequenced characterized amplified region
RAPD: random amplified polymorphic DNA
PCR: polymerase chain reaction
PFGE: pulsed-field gel electrophoresis
HUSM: Hospital Universiti Sains Malaysia
INFORMM: Institute for research in molecular medicine
